# Neutrophilic Myocarditis: Insights from a Forensic Centre’s Retrospective Study

**DOI:** 10.3390/diagnostics14141527

**Published:** 2024-07-15

**Authors:** Oana Neagu, Lăcrămioara Luca, Maria Bosa, Alina Tița, Mihail Constantin Ceaușu

**Affiliations:** 1 Department of Pathology, University of Medicine and Pharmacy Carol Davila, 050474 Bucharest, Romania; 2 Emergency Hospital for Children Grigore Alexandrescu, 011743 Bucharest, Romania; 3 National Institute of Legal Medicine Mina Minovici, 042122 Bucharest, Romania; 4 National Institute of Endocrinology C.I. Parhon, 011863 Bucharest, Romania

**Keywords:** myocarditis, sepsis, positive-air-pressure ventilation, neutrophilic cardiac inflammation

## Abstract

Background: Neutrophilic myocarditis often stems from bacterial or fungal infections, and it is typically detectable through blood cultures or analyses of the primary infection site. However, research specifically addressing the morphological features of acute myocarditis in complex sepsis cases is scarce, with existing studies primarily dating back to the pre-antibiotic era. Methods: This study constitutes a retrospective and descriptive analysis encompassing 22 forensic cases. We collected data from forensic reports emphasising clinical details, disease history, gross observations, and histopathological findings. Results: The results show that using positive-air-pressure ventilation could be related to cardiac inflammation (45.45%, 10/22). Despite large-spectrum antibiotic therapy, the blood samples were positive for Staphylococcus aureus (MRSA strain), Klebsiella pneumoniae (ESBL strain), Acinetobacter baumannii, and Pseudomonas aeruginosa. Colonies developed in the myocardium of 36% of the patients (8/22), where 4 of them had septic emboli. Fungal myocarditis accompanied bacterial infections (2/8) and were unsuspected clinically. Background changes, such as interstitial fibrosis and arteriosclerosis, were associated with a greater degree of inflammation and septic embolism. Conclusion: Neutrophilic myocarditis in patients with emerging sepsis is linked to fatal virulent infections, where bacteria and/or fungi contaminate and impair the myocardium syncytium. Prolonged hospitalisation and positive-air-pressure ventilation may be a risk factor for this condition and needs further research.

## 1. Introduction

Myocarditis is a disease that affects the cardiac muscle and can present with a wide range of clinical symptoms. It can cause mild dysfunction or, in severe cases, lead to acute congestive heart failure, arrhythmias, or sudden death. While viral infections are considered the primary cause, bacterial, parasitic, and non-infectious myocarditis cases have also been reported [1]. Confirming the diagnosis in clinical practice is often challenging as it requires extensive additional testing, including invasive procedures. The gold standard for diagnosis, which involves endomyocardial biopsy, is only available in specialised centres with expertise in interventional cardiology. Consequently, the true incidence of myocarditis is difficult to determine and is likely underestimated. In cases where the underlying cause cannot be confirmed, treatment is typically focused on managing symptoms and providing supportive care until cardiac function improves [2]. However, a comprehensive investigation is necessary to rule out other potential conditions that may benefit from specific therapies.

Myocarditis is characterised by inflammation of the myocardium, which involves necrosis and/or degeneration of heart muscle cells without any damage being caused by reduced blood flow due to coronary artery disease (as defined by the Dallas criteria) [3]. Forensic pathology plays a crucial role in diagnosing and examining the morphological changes associated with myocardial inflammation. During autopsies, cardiac muscle samples are routinely collected and offer valuable insights into the histology of the heart at the time of death. Often, myocarditis goes undetected in clinical settings, and the diagnosis is only established after microscopic examination of the tissue samples. However, based on cellular composition and distribution of the inflammatory infiltrate alone, it is not possible to determine the specific causative factor, such as whether it is viral, bacterial, or non-infectious, in nature. Additional tests, such as PCR tests performed on fresh-frozen cardiac tissue, blood cultures, or serology for antibody detection, are needed to provide further information on the underlying aetiology [4].

Myocardial dysfunction can also occur as a complication of systemic conditions. Previous studies have shown that 40% of patients with sepsis develop cardiac impairment, and the reported mortality rate in these cases can be as high as 70%. While sepsis-induced myocardial suppression often leads to fatal outcomes, it is believed that moderate sepsis can actually result in lower myocyte metabolism, which may help prevent stress-induced cardiac injury [5,6,7]. Sepsis-related myocarditis is commonly caused by bacterial or fungal infections, and the infectious agent can be identified through blood cultures or analyses of the primary infection site. However, there have been very few studies to date that have specifically focused on the morphological characteristics of acute myocarditis associated with complicated sepsis, with most of the existing studies dating back to the pre-antibiotic era [8,9,10]. Some of the common findings reported in case studies include neutrophilic exudates or mixed acute and chronic inflammatory infiltrates, abscess formation, and myocyte necrosis [11]. Neutrophilic myocarditis is a rare histological form typically associated with bacterial myocarditis, and it is most often seen in immunocompromised individuals. It is characterised by the predominance of neutrophils in the inflammatory infiltrates, which can be patchily distributed around myocytes or form micro-abscesses. This condition is usually linked with significant myocyte damage. Various bacteria can cause neutrophilic myocarditis, and this is often caused through the haematogenous spread during septicaemia. It may also arise from the spread of a severe bacterial pneumonia or, less commonly, as a complication of bacterial endocarditis [12].

In this present study, our objective was to identify and characterise all the cases of acute neutrophilic inflammation of the myocardium that resulted in forensic-related deaths.

## 2. Materials and Methods

This retrospective study was carried out at the National Institute of Legal Medicine. We evaluated cases of myocarditis that were identified during post-mortem examinations conducted by forensic doctors. We analysed the database covering a period of five years (January 2015–December 2019), which included a total of 11,660 cases. Only cases with histologically confirmed acute neutrophilic inflammation of the myocardium were included in this study. We retrieved 22 cases with variable morphologies of the inflammatory infiltrate in the myocardium. Information regarding the clinical history, prior symptoms, and circumstances of death was collected from the forensic reports that were available at the time. All the autopsies were performed by specialised forensic doctors following the European protocol [13].

A comprehensive internal examination was conducted, during which detailed photos were taken and tissue specimens were collected. Blood samples were also obtained for toxicology analysis to investigate the presence of illicit drugs, alcohol, or other medications based on the relevant history. In specific cases where sepsis was suspected, blood cultures were performed to identify the underlying cause. Tissue samples included fragments from the brain, lungs, heart, liver, kidneys, and—when necessary—additional specimens such as skin, spleen, and gastrointestinal tract, as determined by the examining physician. One cardiac sample was routinely taken from either the left ventricle free wall or the interventricular septum when no cardiopathy was clinically or grossly suspected. Additional samples were included as required by the forensic pathologist. All tissue specimens were fixed in a 10% neutral buffered formalin solution and sent to the pathology department. The pathologist prepared the tissue samples, selecting specific areas for microscopic evaluation. After processing and paraffin embedding, the tissue blocks were sectioned at a thickness of 3 micrometres and stained with the standard haematoxylin–eosin kit. The histological analysis was performed by an experienced forensic pathologist. 

The final diagnosis of the acute myocarditis was determined based on the criteria established by the Dallas classification [3]. In addition, an auxiliary classification of myocardial inflammation was employed to assess the extent of cardiac damage. To achieve this, we thoroughly examined the myocardial slides and quantified the extent of myocardial inflammation by assessing the percentage of medium-power fields (10× objective) containing inflammatory foci among the entire section examined. All collected data was entered into an anonymized worksheet (Microsoft^®^ Excel^®^ 2019 MSO Version 2406 Build 16.0.17726.20078).

## 3. Results

During the selected five-year period, out of the 11,660 autopsies conducted, a total of 22 cases were histologically confirmed to have neutrophilic myocarditis, accounting for 0.18% of all autopsies. The primary reason for requesting a forensic post-mortem investigation in these cases was to determine the circumstances surrounding a violent or unknown cause of death. The age range of the affected individuals was broad, with the youngest patient being only 4 days old and the oldest patient being 82 years old. The median age of the patients was approximately 41.5 years, indicating that the majority of cases occurred in later adulthood. There was a slightly higher number of male cases compared to female cases, with a male-to-female ratio of 1.44.

According to the clinical files, within this series of cases, prolonged hospitalisation due to trauma resulting from traffic accidents or assaults accounted for 31.8% (7/22). Severe burns were observed in 18% (4/22) of cases, while overdose or complications related to drug addiction were noted in another 18%. Additionally, acute myocarditis was associated with other conditions such as long-term haemodialysis, prosthetic heart valves, congenital heart defects, and stroke. A significant majority of these cases (16 out of 22) shared a common factor of prior hospitalisation, ranging from 1 to 50 days, with a median duration of 11.5 days prior to death. Only one patient had a medically documented episode of ventricular tachycardia, with an irregular rhythm ranging from 135 to 145 beats per minute two hours prior to death. This was followed by ventricular fibrillation, which proved unresponsive to resuscitation efforts. Positive airway pressure (PAP) ventilation was utilised in almost half of the cases (10 out of 22) for a minimum duration of 72 h, as indicated in the clinical records. In hospitalised patients, multisystem organ failure was attributed to the severe sepsis resulting from complicated or unresponsive primary infections in different areas of the body. Despite the administration of broad-spectrum antibiotic therapy, some blood cultures remained positive. The pathogens identified in the post-mortem blood samples included Staphylococcus aureus (MRSA strain, 4/22), Klebsiella pneumoniae (ESBL strain, 2/22), Acinetobacter baumannii (2/22), and Pseudomonas aeruginosa (2/22). Notwithstanding the absence of clinical myocarditis diagnoses, histological examinations indicated that the most severe neutrophilic inflammation was observed in cases with positive blood cultures for Staphylococcus aureus, specifically the MRSA strain. All cases with a known history of drug addiction tested positive for HIV and HCV (4/22), and one of them additionally had tuberculosis.

The histopathology reports revealed that nearly 60% of cases (13 out of 22) exhibited pulmonary infections, primarily presenting as bronchopneumonia with abscess formation. The remaining slides of lung parenchyma showed changes that were attributed to ventilation, pulmonary oedema, or chronic congestion. Kidney involvement was observed in 40% of cases (9 out of 22), displaying features consistent with acute pyelonephritis. Regarding brain microscopy, abnormalities in cerebral tissue were observed in only five cases, including acute encephalitis, ischaemic injury, and haemorrhage. Three cases presented with subarachnoid haemorrhage, while the remaining cases showed only congestion and oedema in the brain.

The cardiac slides underwent a detailed microscopic evaluation to assess the characteristics of the inflammatory infiltrate, including cellularity, distribution, and the presence of any infectious agents such as bacterial colonies or fungal elements. Additionally, the degree of myocardial fibrosis, arteriosclerosis, cardiac myocyte hypertrophy, or sequelae of myocardial infarction was also documented. Among the cases in this series, only two (9%) were associated with endocarditis.

**Figure 1:**
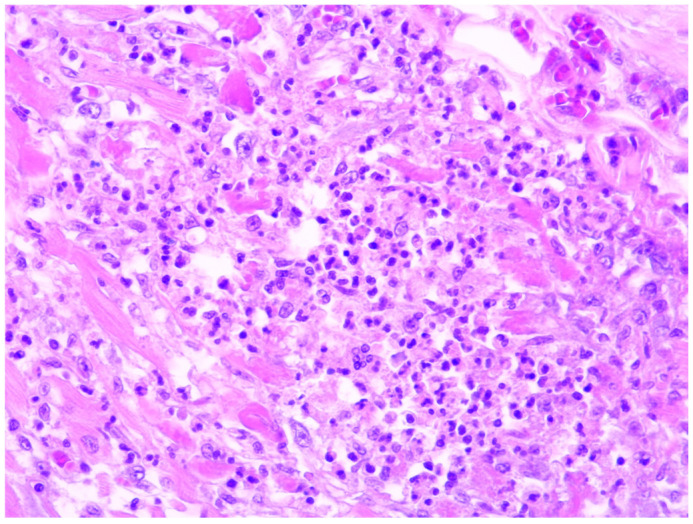
A sample of acute myocarditis focus composed of neutrophils, macrophages, and a few plasma cells (HE, 400×).

**Figure 2:**
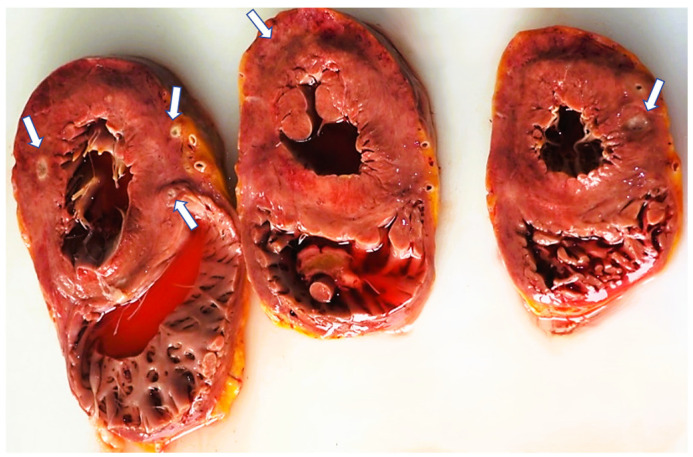
Cardiac transverse sections showing multiple white-yellowish abscesses (white arrows) in the left ventricular wall.

In all our cases, microscopic evaluation revealed a mixed inflammatory infiltrate dominated by neutrophils, and this was followed by macrophages, plasma cells, and a smaller number of lymphocytes (refer to Figure 1). In 17 of the cases, myocarditis was characterised by dispersed, small foci, comprising less than 15% of the entire section and corresponding to grade 2 inflammation severity. Notably, no specific changes in the myocardium were observed during the gross examination at the autopsy. The inflammation was more pronounced in only five cases, occupying between 20 to 40% of a section. In these particular cases, the forensic doctor noticed dark-coloured areas, changes in consistency, or yellowish dots while examining the myocardial sections macroscopically (Figure 2). However, these findings did not raise suspicion of myocarditis at that time.

**Figure 3:**
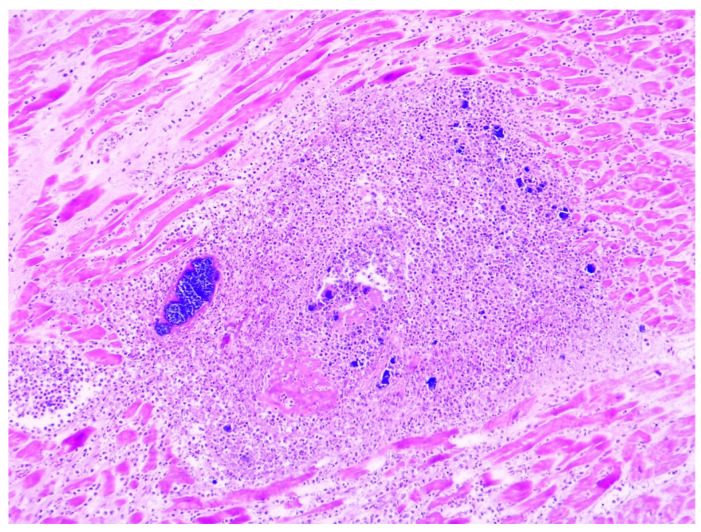
A sample of myocarditis with bacterial colonies (HE, 100×).

**Figure 4:**
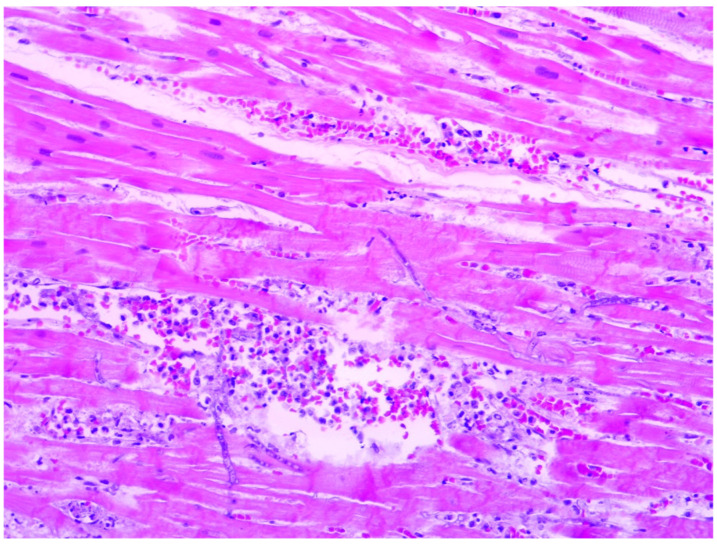
A sample of fungal myocarditis foci showing acute inflammation associated with Aspergillus septate hyphae (HE, 200×).

In 36% of cases (8 out of 22), myocarditis foci were associated with bacterial colonies or fungal elements, as depicted in Figure 3 and Figure 4. Among these cases, septic microemboli were identified within the heart vessels in half of them (4 out of 8). It is worth noting that fungal infection was not previously documented in the clinical files, indicating an unexpected finding. Furthermore, these patients had polymicrobial sepsis, as evidenced by positive blood cultures for Klebsiella pneumoniae (ESBL strain), Pseudomonas aeruginosa, or Staphylococcus aureus. 

Background changes in the heart tissue, such as myocyte hypertrophy and interstitial fibrosis, were observed in 80% of cases (18 out of 22). It was noted that the degree of myocardial fibrosis was higher in cases exhibiting more pronounced myocarditis. Additionally, arteriosclerosis was identified in 3 out of 4 cases that presented with septic emboli.

## 4. Discussion

In the published literature, acute neutrophilic inflammation of the cardiac muscle is commonly linked with overwhelming bacteraemia, frequently as a secondary complication of endocarditis [11,14]. However, in our series, less than 10% of cases showed valve vegetations, suggesting that endocarditis was not the main cause. The pathogenesis of this entity is believed to involve severe bacteraemia, which contributes to the formation of organ abscesses, including the myocardium.

In previously reported studies, sepsis-related myocarditis has been associated with various clinical contexts, including solid organ and hematopoietic stem cell transplantation, chemotherapy, high-dose glucocorticoid use, and human immunodeficiency virus (HIV) infection in patients with acquired immunodeficiency syndrome (AIDS) [15,16]. However, in our study, the primary clinical setting for sepsis-related myocarditis was severe injuries requiring intensive care, particularly trauma or burns over 30% of the total body surface area (TBSA). Only three cases in our series were associated with an immunocompromised status, as these patients were drug abusers and had co-infections with hepatitis C virus (HCV) and HIV. Therefore, the source of infection in the majority of cases was typically a skin injury resulting from trauma, burns, or intravenous drug administration.

Sepsis is often exacerbated by an inappropriate immune response, leading to multiple organ failure and shock. Disseminated abscesses are commonly observed in sepsis and tend to affect various organs, with the kidney, lungs, brain, liver, and adrenal glands being commonly involved in decreasing order of frequency [8]. In this series, the lungs were the most-frequently affected organ, with acute inflammation observed in 60% of cases (13 out of 22), primarily presenting as bronchopneumonia. It is noteworthy that a majority of these patients (10 out of 13) received positive airway pressure (PAP) ventilation for at least three days, which could have potentially influenced their local immune response. The kidney was the second-most-commonly affected organ, observed in over 40% of cases (9 out of 22), and these cases exhibited features consistent with acute pyelonephritis.

The involvement of the heart tissue in sepsis-related myocarditis is still a matter of debate, with various theories proposed by different authors [17]. Previously, it was suggested that cardiac involvement could occur either at the site of a past or recent myocardial infarction [18,19]. However, more recent studies and opinions support the idea that it is primarily a consequence of bacterial seeding of the cardiac muscle [20]. Our findings align with the latter hypothesis as we predominantly observed small foci of inflammation (77%, i.e., 17 out of 22) scattered throughout non-infarcted cardiac tissue, and this was accompanied by septic emboli within the coronary arteries. In rare cases, acute bacterial myocarditis can also present as diffuse inflammation without discrete abscess formation [21,22,23].

The most common causative organism of acute inflammation in the heart is Staphylococcus aureus, although other species such as Streptococcus, Klebsiella, Listeria, Leptospira, and Brucella have also been found in patients with severe sepsis [20]. In our study, the identified pathogens causing bacteraemia were Staphylococcus aureus (MRSA strain), Klebsiella pneumoniae (ESBL strain), Acinetobacter baumannii, and Pseudomonas aeruginosa, which were detected in five cases. Additionally, Candida and Aspergillus were microscopically identified in two rare cases of myocarditis. Candida albicans is typically associated with severe infective endocarditis in heroin addicts [24], but in our case, the patient was immunocompetent and suffered severe burns without evidence of infective endocarditis or other infected foci. The presence of hyphae in the histological examination indicated a superinfection. The second fungal pathogen, Aspergillus, is known to cause invasive aspergillosis, and myocardial involvement is a rare manifestation that usually has a fulminant clinical course [25,26]. In our case, the fungus was exclusively present in the heart without valve infection. The patient also had bacteraemia with Klebsiella pneumoniae, Pseudomonas aeruginosa, Acinetobacter baumannii, and Enterococcus faecium, thus highlighting the importance of considering mycosis in patients with severe sepsis who may benefit from antifungal therapy.

Histologically, sepsis-associated myocarditis often presents as multiple, small, and dispersed foci of acute inflammation that can be easily missed during gross examination [22]. In our study, these small lesions were observed in 77% of cases, involving less than 15% of the cardiac section. Microscopic examination confirmed the bacterial or fungal aetiology in 36% of cases with the presence of bacterial colonies and/or hyphae. However, the remaining cases showed similar patterns of inflammation in terms of cellularity and distribution, indicating a non-viral infection. The identification of septic emboli within the heart vessels further supported the concept of sepsis seeding in the myocardium. Additionally, background changes such as arteriosclerosis were associated with embolism, and the lesions appeared larger in cases with increased interstitial fibrosis, which is consistent with the existing literature [27,28].

## 5. Conclusions

Neutrophilic inflammation of the cardiac muscle can occur in patients treated in intensive care units, even in the absence of an immunocompromised state. The majority of cases (16 out of 22) had a common factor of prior hospitalisation, ranging from 1 to 50 days, with a median duration of 11.5 days before death. Positive airway pressure (PAP) ventilation was utilised in almost half of the cases (10 out of 22) for a minimum duration of 72 h. Disseminated infection leading to organ failure was commonly observed in the lungs, kidneys, liver, and brain. In this retrospective research, the prevalence of bacterial myocarditis was calculated at 0.18%, which aligns with previous studies that have reported myocardial involvement in the form of acute neutrophilic myocarditis with a prevalence ranging from 0.2% to 1.5% at post-mortem [14,22]. While Staphylococcus aureus is the typical infecting organism, other bacteria such as Klebsiella pneumoniae, Pseudomonas aeruginosa, Acinetobacter baumannii, and Enterococcus faecium can also be implicated. In cases of severe sepsis caused by multiple bacteria, the condition may be further complicated by a fungal infection, often involving the Candida species or Aspergillus. Microscopic evaluation of the cardiac tissue revealed a mixed inflammatory infiltrate consisting of neutrophils, macrophages, plasma cells, and a few lymphocytes. Inflammation was typically dispersed in small foci throughout the myocardium. Myocyte hypertrophy, myocardial fibrosis, and arteriosclerosis were observed in a majority of cases.

This study suggests that neutrophilic myocarditis is primarily a consequence of the bacterial seeding of the cardiac muscle, which often results from skin-infected injuries such as trauma, burns, or drug administration. Given the low incidence of endocarditis in our cohort, we can conclude that myocardial seeding can happen more frequently than suspected and could be influenced by highly intensive care therapy, such as positive-air-pressure ventilation (especially in patients with pulmonary infections). This association should prompt a more restrictive therapeutic approach to invasive ventilation to mitigate the risk of myocardial damage. The immune response in sepsis-related myocarditis may contribute to multiple organ failure and shock. Despite the significant degree of myocardial damage, the forensic pathologist did not determine bacterial myocarditis as the cause of death. Given the context of these patients, who had multiple severe organ dysfunctions, it is almost impossible to determine the most critical and, therefore, immediate cause of death. However, this issue was beyond the scope of our research due to limited data and the legal context. Recent research in the field of forensic medicine suggests that the degree of myocardial necrosis and inflammation should be a major criterion in determining whether myocarditis was the cause of death or merely an incidental finding [29,30,31]. Extensive research in forensic medicine could help clarify the hierarchy of pathologies in such complex cases.

In summary, our study highlights the differences between clinical myocarditis and forensic myocarditis. Clinical myocarditis is usually due to lymphocytic inflammation, and it is mainly caused by viral infections. In contrast, neutrophilic cardiac inflammation is rarely detected before death and is usually associated with endocarditis. Moreover, our retrospective study detected a broader and more versatile clinical context for neutrophilic inflammation. Most histologically confirmed myocarditis cases were not clinically suspected, although, in a few cases, myocardial infarction was considered due to elevated cardiac enzymes. Our findings suggest that cardiac impairment in patients with bacteraemia or prolonged hospitalisation can be attributed to the myocardial damage caused by neutrophilic inflammation. Even if blood cultures are sterile and the valves are normal, this does not exclude the possibility of myocarditis. This forensic retrospective study underscores the critical importance of exercising caution when utilising positive-air-pressure ventilation and ensuring a sterile environment for critically ill patients with skin defects or compromised immune responses. These measures are essential for preventing the cardiac impairment caused by insidious bacterial and fungal infections.

## Data Availability

Data available on request due to restrictions (legal reasons).
